# Electricity in Dental Practice

**Published:** 1896-05

**Authors:** L. E. Custer

**Affiliations:** Dayton, Ohio.


ARTICLE V.

### ELECTRICITY IN DENTAL PRACTICE.

BY DR. L. E. CUSTER, DAYTON, OHIO.

The ever-increasing field for the application of the various forms of electricity in dental practice makes it necessary that the dentist should become familiar with the common electrical terms and the different commercial currents in general use.

There are three terms which are the foundation for electrical calculations, the volt, ampere and Watt.

The volt is the term for pressure, and we speak of it just the same as we do of water in a pipe at so many pounds pressure. The ampere is the term for quantity, and to use the same illustration, the ampere represents the carrying capacity of the pipes or cross section of the stream of water-flowing. The Watt is the product of the volts multiplied by the amperes; that is, the quantity of water which flows through the pipe is equal to the size of the opening multiplied by the water pressure. In other words, the Watt is the unit of electric power, and 746 Watts are equal to one horse-power. It does not matter how the Watts are made up, whether more of volts or of amperes, so long as the product of the pressure (volts) multiplied by the quantity (amperes) is equal to 746, it is one horse-power. A current of 75 amperes flowing under a pressure of 10 volts will do work equal to about one horse-power, or a current of one ampere at 746 volts will produce one horse-power. A 16-candle power lamp consumes 55 Watts, at 110 volts that would be half an ampere each, and 13 such burning at one time represents an expenditure of one horse-power. Or a current of 10 amperes at 110 volts is equal to 1,100 Watts, or one and one-half horse-power.

There are five different currents in common use. The arc light current, the 500 volt or car current, the 220 volt or power current, the 110 volt constant or Edison current, and the 52 volt alternating or Westinghouse current.

The arc current is familiar to all, and it affords an interesting example in electric arithmetic. It requires a pressure of 45 volts to leap across the distance between the two carbons of the lamp to give the light we see, but to give a steady light there should be 10 amperes of current. These 10 amperes of current are started out from the power house, and for every arc lamp through which they pass there is required an addition of 45 volts. That is, a pressure of 45 volts is required to jump the arc and maintain it in the first lamp. This lamp mnst be kept burning, and when the second lamp is reached, the dynamo quickly makes an additional 45 volts, and so on all the way around the circuit back to the power house. So that, if there are 50 arc lamps in the circuit there would be required 50 times 45, or 2,250 volts to send a current of 10 amperes through the 50 lamps. The high voltage of the arc current is the cause of the danger. It is volts that kill.

The 500-volt current is used mostly for street cars and heavy power work. The high voltage is used here for the same reason that it is more economical to carry water in small pipes at high pressure than in large pipes at low pressure, as well as the fact that the motors are better proportioned for their work when operated by this current. It will be noticed that these cars are always lighted by five 110 volt lamps in series; that is, it requires 110 volts to properly burn one lamp, but with 500 volts pressure the current can go through one and then another till it has passed through five lamps, and each one will burn but little below its intended capacity. If four lamps were used they would soon burn out, because there would be 125 volts to a lamp instead of 110.

The 220-volt current is mostly used for power purposes, because it can be carried on a rather small wire and is not especially dangerous to life. This current is not, however, very common.

The Edison or 110-volt current is, as you well know, almost universally used for incandescent lighting and light power. This current does not differ from any of the preceding except as to voltage. It is sometimes called the constant current because the current is continually flowing in one direction. The pressure is set at 110 volts because it is found to be economically distributed at this voltage to that point where the voltage drops below 100. For this reason it is always used in small plants, or in large plants whose wires do not run far from the power house, in thickly settled centers, and in hotels and public buildings.

The 52-volt or alternating current is used for incandescent lighting and light power also. This differs from the preceding in many ways. Instead of its flowing in one direction it flows alternately in one direction and then in the other. This current is distributed in an entirely different manner from the others. A current of very high voltage is conducted to what is known as the transformer, passing through which it induces an extra current in an entirely independent coil of wires, and this new or induced current is the one carried into the house for use. So that the primary current of very high voltage is carried on a comparatively small wire for miles about the city at quite a small expense, and gives the consumer a light which would be impracticable with a constant current at that distance from the power station. This current is used mostly in small towns and in scattered suburbs.

Of these currents, the Edison or 110 volts constant potential current, is the ideal one for dental purposes and fortunately it is the most common. It is so easily made that nearly every office building operates its own plant. Or the dentist with a gasoline engine and dynamo can be independent at a very small cost. The current is safe to life and its wiring is quite simple. It may be used for power, for light, for cataphoresis, for electrolysis and for heat equally as well. In fact, it seems as though this current was intended for dental purposes alone. Let the dentist be a little inventive, and he will see many applicants of electricity that will be of great benefit.—*Dental Review.*

